# Medical and Philosophical Intelligence

**Published:** 1812-06

**Authors:** 


### MEDICAL AND PHILOSOPHICAL INTELLIGENCE.

ROYAL SOCIETY.—*April* 9th and 16th.—A paper was read on arsenic, by Dr. Lambe, of King's Road, Bedford Row, communicated by the late Dr. Garthshore.

The principal object of Dr. L.'s labours on arsenic seems to have been to defend an hypothesis proposed by him, a considerable time ago, in two successive publications, namely, "An Inquiry into the Origin of Constitutional Diseases," and "Reports on Cancer," in which the doctor attempted to show the analogy between the action of putrid matter and arsenical poison. The facts of most consequence, in the paper read as above to the Royal Society, are the following:

1. One part of charcoal was mixed with ten of white arsenic and twenty of nitre: after deflagration, no signs of carbonic acid could be detected, as would have happened had no arsenic been present.

2. Small quantities of charcoal mixed with white oxide of arsenic had the same effect as upon many other metallic oxides, producing carbonic acid and carbonic oxide.

3. In the reduction of arsenic by mixing white arsenic, half its weight of subcarbonate of soda, and $\frac{1}{6}$th part of charcoal, there appeared a peculiar gas in the early stage of the process. It could not be inflamed by a candle, (mixed either with oxygen or common air,) but was inflammable by electricity, and was thus convertible into carbonic acid, and a considerable portion of azote remained after after the inflammation. The properties of this gas changed by being kept in a phial uncorked: the inflammable portion diminished, and the residuum of azote increased.

After this gas had come over, large portions of hydrogen and carburetted hydrogen were obtained. They were mixed with small portions of azote and oxygen to the end of the process.

Besides these gases there was also carbonic acid in great abundance.

4. These experiments were performed in earthen retorts, which might be suspected to permit some atmospheric air to pass through their pores: and such appears on trial to be the case. For, on using glass retorts, the results were considerably different. The first products were carbonic acid, and small quantities both of azote and oxygen, which were left after the carbonic acid had been removed by lime-water. But towards the end of the process, instead of a residuary portion of azote and oxygen, a peculiar gas was observed similar in many respects to that described. It could not be inflamed by a candle, when mixed with atmospheric air, or with oxygen gas; but it inflamed by a smart electric shock, and carbonic acid was produced. A more minute research detected also the production of nitric acid from the detonation of this gas. On this account Dr. Lambe has given it the name of *nitro-carbonic oxide.*

5. When this process was conducted in glass retorts, not the smallest vestige of hydrogen or carburetted hydrogen could be observed, which were so abundant when the earthen retorts were used. But still small quantities both of azote and oxygen came over to the very end. Water also appeared to be produced in every stage of the process.

---

*The Kirwanian Society of Dublin.*—A new Philosophical Society has been established in Dublin, on a plan somewhat different from those already existing in that city. Its object is, to promote the cultivation of chemistry, mineralogy, and other branches of natural history; and it means to concentrate its attention to these pursuits exclusively.

The members, desirous of paying the greatest and only tribute of respect in their power to the venerable and illustrious Mr. Kirwan, long distinguished in the first rank of philosophers, for a long continued course of labors equally useful to the world and creditable to himself, have resolved upon establishing themselves under the name of the KIRWANIAN SOCIETY.

The society, except during the summer vacation, holds its stated meetings once every fortnight.

Communications on subjects connected with the above sciences will be received and read to a meeting of the society: for this purpose, a meeting will be even summoned at any time during the summer vacation. The more effectually to ensure the right of anteriority to any original communication, the day of its receipt will be registered in a book kept for that purpose.

The society will gratuitously circulate concise printed directions, for the selection of such important or rare minerals as may promote the progress of mineralogical knowledge. These directions shall be particularly calculated for drawing the attention of the less experienced to such specimens as indicate the vicinity of metallic veins or other mineral treasures.

Any person having in his possession minerals, with the nature of which he is unacquainted, may have them examined, or, if of sufficient importance, even analysed, by transmitting them to the secretary of the society.

A set of instruments for mineralogical observation or research will be always kept in readiness by the society, for the use of those who have not such already in their possession, and who may be deemed likely to pursue such inquiries with advantage.

Such are the present objects of the society, and they will no doubt in time be extended so as to become more generally useful. A fuller statement will, we understand, be shortly published in a Prospectus.

---

Messrs. Clements and Desormes, in France, have improved upon the experiments of Professor Leslie, to produce ice by evaporation in the air-pump. They have proposed to apply the evaporation, in vacuo, on a large scale, to the drying of gun-powder; which being affected without fire, is attended with no danger. The French chemists also are engaged in experiments to apply the evaporation in vacuo, to the drying and preserving fruits and vegetables.

Professor Leslie, however, claims the discovery, and we doubt not that very important results will be obtained by it. He has lately succeeded, at Edinburgh, in freezing quicksilver by his frigorific process. This curious experiment was performed with an air-pump on a new and improved construction, made by Mr. Adée, the optician. A wide thermometer tube, with a large bulb, was filled with mercury, and attached to a rod passing through a collar of leathers, from the top of a cylindrical receiver, seven inches wide. It covered a deep flat bason of nearly the same width, containing sulphuric acid, in which was placed an egg-cup half full of water. The inclosed air being reduced by the working of the pump to the 50th part, the bulb was repeatedly dipt in the water, and again exposed to evaporation, till it became incrusted with a coat of ice about the 20th of an inch thick. The cup, with its water still unfrozen, was then removed, and the apparatus replaced, the coated bulb being pushed down to less than an inch from the surface of the sulphuric acid. On exhausting the receiver again, and continuing the operation, the icy crust at length started into divided fissures, owing probably to its being more contracted by the intense cold than the glass which it invested; and the mercury, having gradually descended in the thermometer tube till it reached the point of congelation, suddenly sunk almost into the bulb, the gage standing at the 20th of an an inch, and the included air being thus rarefied about 600 times. After a few minutes, the apparatus being removed, and the bulb broken, the quicksilver appeared a solid mass which bore the stroke of a hammer. The temperature of the apartment was then 54° of Fahrenheit. In another experiment, with a small spirit of wine thermometer, under the same circumstances, and the same degree of rarefaction, the cold produced was found to be $70\frac{1}{2}$° below nothing, or more than 30° below the point usually assigned for the congelation of mercury.

---

*Case of Injury of the Head, by John Burne.*—J. T. aged 36, for the last ten months has been a constable. About five months ago he was struck on the forehead by a man he was endeavoring to secure. He felt very little inconvenience from the blow, and went about his employment as usual.

Jan. 3, 1812. He said he was very unwell, with a dull heavy pain in the head, but he complained of nothing else; his appetite was very good; his bowels pretty regular; his eye-sight very good; and he could walk about quite well.

℞. Pulveris Rhei.

Terebinthinæ Chiæ aa ℥j. M. Ft. pilulæ xxx. quarum duas bis die sumat.

℞. Emplastrum Picis compositum Nuchæ.

Jan 7th. He found himself much better; the pain was nearly gone, and he thought he should have no need to apply again. The above treatment continued.

Jan. 17th. I have heard nothing of him since the 7th till this morning, when he sent to request that I would visit him, for he was confined to his bed, and very dangerously ill. When I visited him (which was about noon), I was informed from his wife that he had continued to get much better till the 16th, when he was suddenly seized with pain in the head, giddiness, trembling; lost his speech. and the use of his right arm. In this state I found him; the light did not affect his eyes; he seemed very dull and stupid; his bowels were confined.

℞. Submuriatis Hydrargyri gr. vj.
Pulveris Rhei gr. x. M. Ft. pulvis statim sumendus ex Theriacâ.

℞. Emplastrum Lyttæ Nuchæ.

Jan. 18th. Bowels have been purged; has passed a very restless night; cannot speak; in other respects the same; his skin hot and dry.

℞. Misturæ Camphoræ ℥vj.

Liquoris Ammoniæ Acetatis ℥ij.

Liquoris Antimonii tartarizati ʒij. M. Fiat mistura, cujus sumat cochlearia duo ampla quartis horis.

Jan. 19th. No better; has passed a very restless night; cannot speak; right arm still insensible, and he has no power to move it.

 ℞. Submuriatis

℞. Submuriatis Hydrargyri gr. j.
Pulveris Antimonialis gr. ij.
Nitratis Potassæ gr. xv. M. Fiat pulvis quartis horis sumendus ex Melle.

℞. Emplastrum Lyttæ largum toto capiti.

Jan. 20th. Somewhat better; can speak a few words, but very indistinctly; has had no sleep; the light during the whole time has produced no pain or inconvenience to his eyes; there is much stupor and dulness about him; he complains of nothing, but shakes his head when he is asked how he does. The powders continued.

Jan. 21st. Has had a little sleep; can speak rather more distinct; his right arm is sensible to the touch, and he can move it a very little. The same treatment continued.

Jan. 22d. His bowels are regularly open; has passed a better night; moves his right arm rather more freely, and can take a little food. The powders continued, but not so often.

Jan. 24th. Much better; can sit up in the bed and eat some meat; sleeps tolerably well, but has a troublesome tickling cough. The powders are discontinued.

℞. Oxymellis Scyllæ.
Syrupi Papaveris albi aa ℥j. M. Cochleare minimum urgenti tusse capiat.

Jan. 30th. Cough relieved; has taken no other medicine; he is able to get up and walk about the room; appetite pretty good; his right arm is more sensible, and he can move it much better; speaks more distinctly. I put a seton in the back part of the neck, and ordered him to take no medicine whatever.

Feb. 12. The seton has discharged copiously, and he has found very great benefit from it; he sits up all day; can walk very well; has quite recovered the use of his arm; appetite very good; sleeps very well, and can speak nearly as well as ever, and now wants nothing but strength to enable him to resume his former employment.

Is it not most probable that the above symptoms were occasioned by effusion causing compression in consequence of the blow, or by inflammation? The symptoms seemed to indicate the former more than the latter.—*(Phil. Mag.)*

---

Amongst the few instances of early puberty on record, the following is not the least remarkable. M. Gangirau had occasion to see (the beginning of June 1810,) a peasant's child called Isabeau Viole, a native of Fonsegrives, three miles from Toulouse. She was aged four years and three months, had menstruated from the age of three years, and the catamenia appeared regularly each month. The canine and incisor teeth had been already renewed, and, according to the mother's report, the first dentition was extremely premature. The sexual organs were furnished with thick down. The breasts were prominent as in a girl of sixteen or seventeen, but not so firm and elastic as is usual at that age. Her height was three feet, feet, ten inches, and six lines. She measured round the waist twenty-three inches, five lines; the complexion was clear brown; she had black eyes; and her constitution appeared to be strong and vigorous.

---

Mr. Stevenson has just concluded a very interesting course of twenty-four Lectures on the Anatomy, Physiology, and Diseases, of the Eye and Ear, which he purposes to repeat during the ensuing winter, at his house in Great Russell-street, Bloomsbury. An opportunity will thus be afforded the student of acquiring a scientific acquaintance with a very important class of diseases.

---

*Medical and Chemical Lectures and Examinations.*—Dr. Hooper and Dr. Ager will begin another course on the Theory and Practice of Physic, Chemistry, and the Materia Medica, at the theatre, 26, Cork-street, Burlington Gardens, on Monday, June 1st, 1812, at eight o'clock in the morning. Three courses are given yearly, beginning in February, June, and October, each on the first Monday of the month.

---

Plan of Dr. Adams's Lectures on the Institutes and Practice of Medicine, to commence on the 9th of June.—In the Institutes will be given a general view of the various Systems of Physiology, ancient and modern, as applicable to Pathology, including the Discoveries of Mr. Hunter, which are too often imperfectly explained, or applied only to Surgery. The Doctrine of Inflammation, the Experiments on which it is founded, and the Importance of its Application in all Acute Diseases, will be minutely dwelt upon. The practical part of the course will comprehend the Application of this knowledge to the Treatment of Diseases Acute and Chronic. Cases which may occur of sufficient importance at the Small-pox and Innoculation Hospitals, or at the Central Dispensary, will be exhibited, with the Progress of Variolation and Vaccination in all their stages. The Therapeutic Part of the course will comprehend a general view of the Materia Medica, as far as is applicable to Practice.

---

Dr. Squire will on Saturday, June 6th, commence a course of Lectures on the Principles and Practice of Midwifery, and the Diseases of Women and Children. Particulars may be known by inquiry at the doctor's house, No. 30, Ely-place, Holborn.

---

*Medical and Chemical Lectures, St. George's Hospital, and No. 9, George-street, Hanover-square.*—On Monday, June 1st, these Lectures will re-commence as usual, viz. the Medical Lecture at eight, and the Chemical at a quarter after nine, in the morning, by George Pearson, M.D. F.R.S. Senior Physician of St.

 George's

George's Hospital, of the College of Physicians, &c.—*Note.* Lectures on the Patients in St. George's Hospital are also given every Saturday morning, at nine o'clock.

---

Mr. Thompson's usual course of Lectures on the Science of Botany, will commence on Friday next, the 29th of May. The lectures will be delivered in the library of the botanic garden, Sloane-street, every morning, except on Saturdays and Sundays, at eight o'clock; and his pupils will have the privilege of examining the specimens in the garden, and every other advantage of the institution. This course is particularly designed for medical students; and the specimens by which the lectures will be illustrated, will be those, chiefly, which are articles of the materia medica. A syllabus and other particulars of the course, may be had by application to Mr. Thompson, at his house, No. 92, Sloane-street.

---

We have this month the painful task of announcing the death of Dr. Willan, in the island of Madeira. Our last advices from that place expressed hopes of his recovery, but his ascites still continuing obstinate, his friends could not but be doubtful of the event. About the middle of March the symptoms increased rapidly, and on the 7th of April he expired. He was interred at the English burial-ground at Funchall.

